# IN THE SHADOW OF THE GREAT RECESSION: THE ASSOCIATION BETWEEN RECESSION EXPERIENCES AND DAILY INDICES OF WELL-BEING

**DOI:** 10.1093/geroni/igac059.2404

**Published:** 2022-12-20

**Authors:** Jonathan Rush, Aarti Bhat, Duncan Thomas, Arun Karlamangla, David Almeida, Teresa Seeman

**Affiliations:** University of Victoria, Victoria, British Columbia, Canada; The Pennsylvania State University, State College, Pennsylvania, United States; Duke University, Durham, North Carolina, United States; University of California, Los Angeles, Los Angeles, California, United States; The Pennsylvania State University, University Park, Pennsylvania, United States; University of California Los Angeles, Los Angeles, California, United States

## Abstract

The current study examined the associations of positive and negative experiences during the Great Recession (GR) with levels of daily well-being. In 2012, participants from the Midlife in the United States Refresher survey reported on their positive or negative GR experiences related to job, housing, or finances. A subsample, selected into the National Study of Daily Experiences (N=782), also reported on their daily levels of health and well-being across eight consecutive days and provided saliva samples, from which cortisol was assayed. The number of negative GR experiences reported related to poorer daily well-being (negative and positive affect, physical symptoms, stress severity, and cortisol daily peak-to-nadir ratio), whereas, the number of positive GR experiences was only related to lower severity of daily stressors (β=–0.03, p=.03). Examining specific GR experiences revealed that individuals who reported bad housing experience during the GR reported higher daily levels of negative affect (β=0.14, p<.001), physical symptoms (β=0.90, p<.001), and frequency of stressor days (β=0.01, p<.001), and lower daily levels of positive affect (β=–0.19, p=.02). Bad financial experience was related to more physical symptoms (β=0.62, p<.001) and greater severity of daily stressors (β=0.14, p=.03). Conversely, positive financial experiences were related to greater cortisol daily peak-to-nadir ratio (β=1.98, p=.03), but also greater frequency of stressor days (β=0.05, p=.01). Results highlight the potential influence of major economic strains on our ongoing daily experiences. This work has implications for policy and interventions around supporting midlife and older adults facing economic strains, in order to improve daily well-being.

